# Supplementary material for: A Theoretical Investigation into the Oligomer Structure of Carbon Dots Formed from Small-Molecule Precursors
Source: Molecules. 2024 Jun 19;29(12):2920. doi: 10.3390/molecules29122920 (PMC11206910; doi:10.3390/molecules29122920)
Supplement: Supplementary file 1 [file molecules-29-02920-s001.zip › molecules-3044705-supplementary.pdf]

Supplementary Information

A Theoretical Investigation into the Oligomer Structure of Carbon Dots Formed from Small-Molecule Precursors

Chunlan Li, Xu Zhu * and Maotian Xu

Henan Key Laboratory of Biomolecular Recognition and Sensing, Henan Joint International Research Laboratory of Chemo/Biosensing and Early Diagnosis of Major Diseases, College of Chemistry and Chemical En-gineering, Shangqiu Normal University, Shangqiu 476000, China; chunlan16@yeah.net (C.L.); xumaotian@sqnu.edu.cn (M.X.)

*Correspondence: ocina@yeah.net

Tables and Figures

Table S1. Lowest frequency and Gibbs free energy of CA and EDA

Species	Frequency/cm^{-1}	Gibbs free energy/hartree
CA	43	-760.239415
EDA	139	-190.517966

Table S2. Lowest frequency and Gibbs free energy of dimeric isomers formed under the weak interaction of CA and EDA in aqueous solution

Isomer	[1]Frequency /cm^{-1}	[1]Gibbs free energy/hartree	[1, a] ΔG /kJ·mol^{-1}	[2]Frequency /cm^{-1}	[2]Gibbs free energy/hartree	[2, a] ΔG /kJ·mol^{-1}
di-IM1-1-1	13	-950.774884	15.0	16	-950.799931	18.2
di-IM1-1-2	29	-950.759430	55.6	21	-950.783220	60.5
di-IM1-1-3	14	-950.774570	15.8	22	-950.797666	22.5
di-IM1-1-4	6	-950.774590	15.8	15	-950.796895	24.6
di-IM1-1-5	12	-950.773911	17.5	21	-950.796897	24.6
di-IM1-1-6	23	-950.765761	38.9	17	-950.794159	31.7
di-IM1-1-7	16	-950.773357	19.0	19	-950.796945	24.4
di-IM1-1-8	33	-950.764401	42.5	23	-950.794266	31.5
di-IM1-1-9	22	-950.764419	42.5	20	-950.793416	33.7
di-IM1-1-10	19	-950.765895	38.6	31	-950.793112	34.5
di-IM3-1+H_2O	21, 1610	-950.780592	0	29, 1589	-950.806244	0

[1] Lowest frequency and Gibbs free energy of dimeric isomers were carried out under the consideration of the conductor-like polarizable continuum model (CPCM).

[2] Lowest frequency and Gibbs free energy of dimeric isomers were carried out under the consideration of solvation model based on density (SMD).

[a] ΔG is the difference in free energy of each isomer relative to "di-IM3-1 + H_2O".

Comparison results: Under the same conditions in the computing environment, the Gibbs free energy obtained using the SMD solvent model is slightly lower than that acquired using the CPCM solvent model, but the decreasing trend of their free energy is basically the same. The conclusions obtained from the two implicit solvent models are consistent: the Gibbs free energy of the dimer product (di-IM3-1 + H_2O) connected by amide bonds is lower than that of other dimer isomers connected by intermolecular hydrogen bonding. CA and EDA will preferentially combine through amide bonds to obtain the product di-IM3-1.

Table S3. Lowest frequency and Gibbs free energy of oligomeric isomers formed under the weak interaction of di-IM3-1 and EDA in aqueous solution

Isomer	Frequency/cm^{-1}	Gibbs free energy/hartree	^{a} ΔG /kJ·mol^{-1}
tri-IM4-1	24	-1064.847157	11.7
tri-IM4-2	27	-1064.833546	47.4
tri-IM4-3	18	-1064.836008	40.9
tri-IM4-4	15	-1064.841595	26.3
tri-IM4-5	24	-1064.835373	42.6
tri-IM4-6	12	-1064.841107	27.6
tri-IM4-7	19	-1064.847103	11.8
tri-IM4-8	18	-1064.838622	34.1
tri-IM4-9	16	-1064.840180	30.0
tri-IM4-10	14	-1064.840513	29.1
tri-IM6+H_2O	17, 1610	-1064.851596	0

^{a} ΔG is the difference in free energy of each isomer relative to "tri-IM6 + H_2O"

Table S4. Lowest frequency and Gibbs free energy of oligomeric isomers formed under the weak interaction of two tri-IM6 molecules in aqueous solution

Isomer	Frequency/cm^{-1}	Gibbs free energy/hartree	^{a} ΔG /kJ·mol^{-1}
hex-IM7-1	10	-1976.795778	4.4
hex-IM7-2	17	-1976.791845	14.7
hex-IM7-3	10	-1976.792836	12.1
hex-IM7-4	18	-1976.789667	20.1
hex-IM7-5	21	-1976.792788	12.3
hex-IM7-6	20	-1976.785277	32.0
hex-IM7-7	17	-1976.791384	15.9
hex-IM7-8	12	-1976.786059	29.9
hex-IM7-9	27	-1976.794799	7.0
hex-IM7-10	14	-1976.794583	7.5
hex-IM7 hex-	16	-1976.797456	0
IM9+H_2O	16, 1610	-1976.792698	12.5

^{a} ΔG is the difference in free energy of "hex-IM9+H_2O" and other isomers relative to "hex-IM7"

Table S5. Lowest frequency and Gibbs free energy of oligomeric isomers formed under the weak interaction of tri-IM6 and EDA in aqueous solution

**Isomer**	**Frequency /cm^{-1}**	**Gibbs free energy /hartree**	**[a] ΔG /kJ·mol^{-1}**
CA-3EDA-IM1-1	14	-1178.916325	15.1
CA-3EDA-IM1-2	21	-1178.912687	24.7
CA-3EDA-IM1-3	23	-1178.912149	26.1
CA-3EDA-IM1-4	13	-1178.915964	16.1
CA-3EDA-IM1-5	33	-1178.906474	41.0
CA-3EDA-IM1-6	22	-1178.911253	28.5
CA-3EDA-IM1-7	26	-1178.906797	40.2
CA-3EDA-IM1-8	19	-1178.910893	29.4
CA-3EDA-IM1-9	23	-1178.914978	18.7
CA-3EDA-IM1-10	11	-1178.909088	34.2
CA-3EDA-IM3+H_2O	12, 1610	-1178.922090	0

[a] ΔG is the difference in free energy of each isomer relative to “CA-3EDA-IM3 + H_2O”

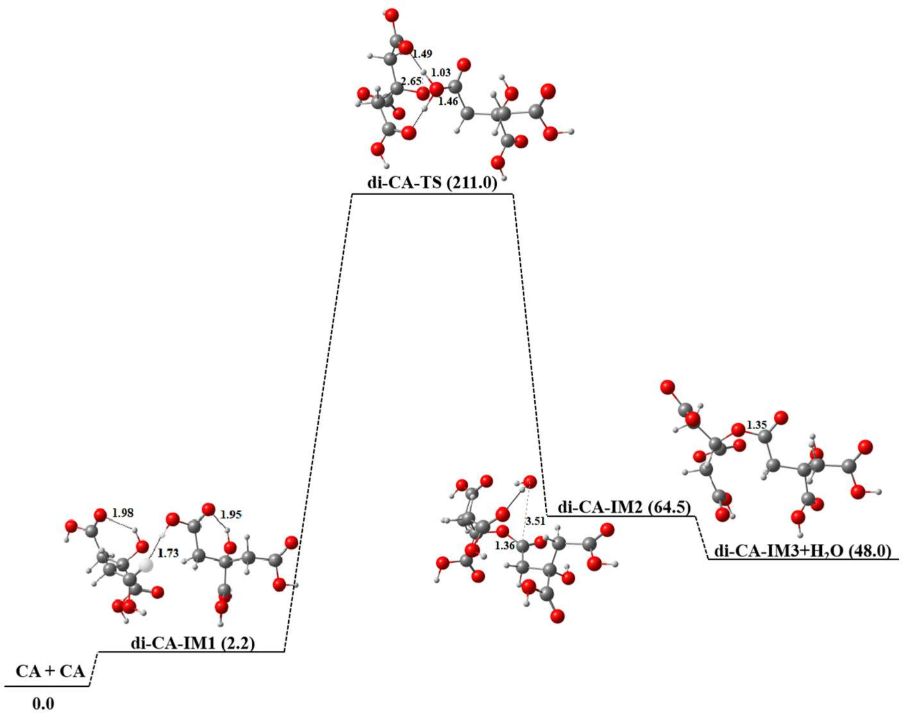

Figure S1. Dimerization of CA in aqueous solution; the relative energies are in kJ·mol^{-1}, this unit is uniformly used in all figures. Note: aluminium in pink, carbon in dark grey, oxygen in red, and hydrogen in light grey.

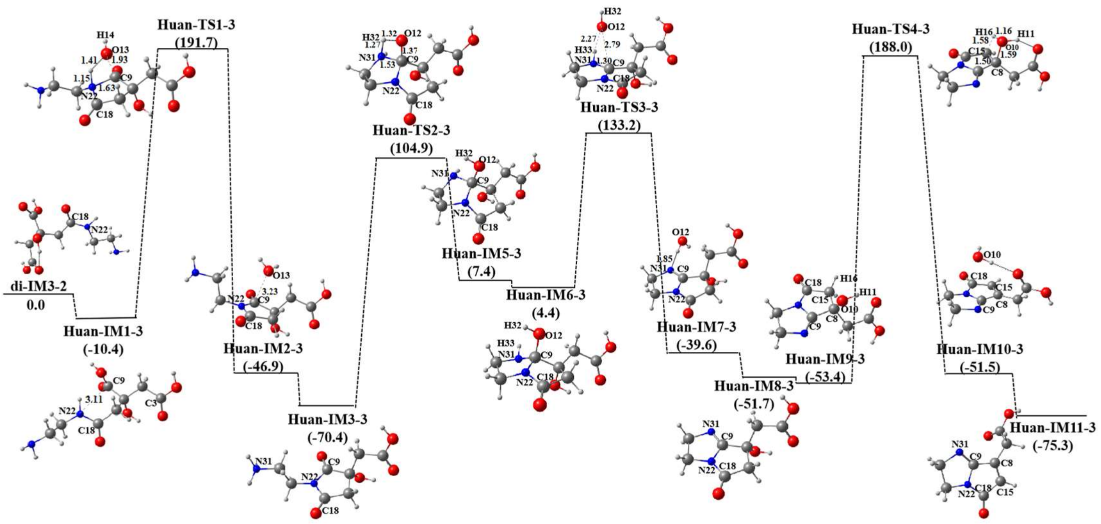

Figure S2. The second cyclization path of di-IM3-2 in aqueous solution
